# Emergence of Leadership in Communication

**DOI:** 10.1371/journal.pone.0159301

**Published:** 2016-08-17

**Authors:** Armen E. Allahverdyan, Aram Galstyan

**Affiliations:** 1 Yerevan Physics Institute, Alikhanian Brothers Street 2, Yerevan 375036, Armenia; 2 USC Information Sciences Institute, 4676 Admiralty Way, Marina del Rey, CA 90292, United States of America; Centre de physique theorique, FRANCE

## Abstract

We study a neuro-inspired model that mimics a discussion (or information dissemination) process in a network of agents. During their interaction, agents redistribute activity and network weights, resulting in emergence of leader(s). The model is able to reproduce the basic scenarios of leadership known in nature and society: laissez-faire (irregular activity, weak leadership, sizable inter-follower interaction, autonomous sub-leaders); participative or democratic (strong leadership, but with feedback from followers); and autocratic (no feedback, one-way influence). Several pertinent aspects of these scenarios are found as well—e.g., hidden leadership (a hidden clique of agents driving the official autocratic leader), and successive leadership (two leaders influence followers by turns). We study how these scenarios emerge from inter-agent dynamics and how they depend on behavior rules of agents—in particular, on their inertia against state changes.

## Introduction

The notion of opinion leaders has become paradigmatic in social sciences [1, 2]. Identifying leaders can be important for applications such as viral marketing, accelerating (or blocking) the adoption of innovations, *etc*. Communication research postulates that informational influence in groups often happens via a two-step process, where information first flows from news media to opinion leaders, and then is spread further to followers [3]. This theory is believed to adequately account for consumer behavior [4] and has been refined in several ways [5, 6].

A significant research has focused on identifying traits and characteristics of leaders. For instance, it has been observed that opinion leaders are *not* necessarily more educated than followers, but they typically have higher income [7]. However, it was understood that no single trait (or even a cluster of traits) can explain the emergence of leadership [8]. It is believed that leaders can be imposed externally, or emerge within the group [9]—possibly out of purely random reasons [10]—together with agents who are not opinion leaders, but are important for ensuring that the leaders function [11].

Social psychology developed several qualitative theories on how leaders perform in groups [12]. The contingency theory—developed in opposition to “leadership trait” approaches— focuses on the nature of interactions between the leader and followers [12–14]. According to this theory, the effectiveness of a particular type of leadership is contingent on the favorability of the situation to that type. Based on earlier research [13], the contingency theory identified several major types of leaders [14]:

– Laissez-faire leadership is characterized by a relatively weak guidance of autonomous followers. It occurs in systems such as scientific collectives.
– Participative (democratic) leaders do influence their followers strongly, but encourage and accept feedback from them.
– No feedback (from the majority of followers) is accepted by autocratic leaders. This type of leadership is typically present in military, businesses and governance systems.

Given the above typology, which is well-confirmed by everyday experience, it is not completely clear which features imply specific leadership types [1, 2]. Our main purpose is to study this problem via a mathematical model.

The network theory developed methods for identifying opinion leaders via the core-periphery (or star) structure of social networks [15–20]. Such structures can emerge from imposing on the network certain functional goals or optimization principles [21–24]. Recent research on social networks proposed several methods for uncovering hidden network of influences [25–29], and identifying core-periphery structures related to opinion leaders [27].

Several models were proposed for describing the leadership phenomenon. Ref. [30] considers game-theoretic models, where the leadership is defined via the possibility of the first move in a game. The related notion of Stackelberg equilibrium is one of the basic game-theoretic manifestations of leadership [31]. Models of adaptive (inhomogeneous) networks identify emergent leaders with well-connected nodes in a network of game-theoretic [32, 33] or resource-distribution units [34, 35]. Other approaches to emergent leadership are reviewed in [36, 37]. The leadership problem relates to diversity modeling in social and biological systems [38, 39]; see [40] for a review.

Despite of much inter-disciplinary research devoted to the leadership issue, we seem to lack a basic model that can reproduce in a single set-up the major leadership scenarios, and relate them to behavior of involved agents.

We intend to provide a formal, mathematical model for the emergence and the type of leadership in a collective of interacting agents modeled via neurons. Certain analogies between human agents and neurons were noted in literature [41–53]. Both neurons and human agents are adaptive entities that form communities, analyze information, and can specialize for different roles and functions in (resp.) brain and society [45]. Quantitative sociology employs neuronal models for describing weak ties [54], social impact [55], and economic activity [56–58]. Neuronal models are capable to generalize on the concept of activity cascade that is frequently employed in social modeling [59–61]. The major limitation of this concept is that the activation of each network node occurs only once. However, there is an ample of empiric evidence that social activity patterns are recurrent [28, 29, 62], an effect well described by neuronal models [52, 53].

Our model mimics a discussion process, where opinion expression by one agent facilitates activation of other agents. Experimental studies on the leadership emergence in discussion groups were carried out in [63, 64]. It was noted there that the leader emerges due to it active involvement into the group dynamics, e.g. due to active talking, while the quality of this talking is not very important (babble effect) [63, 64].

We postulate tractable rules for the agent’s behavior. These rules incorporate major factors that are relevant for the leadership, e.g. activity, attention, initial social capital (i.e. well-connectedness in the network), and score (credibility). The rules depend on parameters that characterize the agent’s “conservatism” with respect to changing its state.

The leader is naturally defined as an agent that influences other agents strongly (i.e. stronger than those agents influence the leader), and that actively participate in the group activity, in the sense that blocking the leader will diminish (or at least essentially decrease) the activity of the group.

Our main result is that the three basic leadership scenarios—laissez-faire, participative and autocratic—emerge under different behavioral rules. Here is a short list of certain specific leadership features predicted by the model.

The laissez-faire leadership emerges under noisy, but score-free dynamics. It relates to irregular activity patterns, allows autonomous sub-leaders and sizable communication between followers. In the participative situation possible sub-leaders are strict subordinates of the leader. A participative leader emerges due to an initial (possibly small) advantage in its social capital. If the inter-agents interaction is sufficiently strong, the emergent participative leader wins over an externally imposed candidate for leadership. The autocratic situation is more vulnerable to an externally imposed leader, than the participative one. There are cases, where the “official” autocratic leader is driven by a hidden click of other agents. Coalitions of autocratic leaders are possible, but they are meta-stable, and for long times reduce to just two leaders driving the followers by turns.

## The model

### State Dynamics

The main ingredients of our model are listed in Table 1. We consider *N* agents. At a given moment of discrete time *t*, each agent can be active—give an opinion, ask question *etc*—or passive. For each agent *i* (*i* = 1, …, *N*) we introduce a variable *m*_*i*_(*t*) that can assume two values 0 (passive) and 1 (active).

**Table 1:** Here we list the main ingredients of the model with relevant notations and equations that introduce them.

Equations and references	Ingredients of the model
*i* = 1, …, *N*	*N* agents modeled as neurons in discrete time *t* = 1, 2, ….
Eqs (1) and (2). Refs. [65–67].	Firing rule for activity *m*_*i*_(*t*) of discrete agents-neurons.
Eqs (4) and (5). Ref. [71].	Constrained network weights *τ*_*ij*_ model attention limitation.
Eq (6).	Adaptation of weights: an active agent gets more attention from others. Parameter *α* accounts for inertia.
Eqs (7) and (9).	An agent which gets more attention obtains larger score *σ*_*i*_. The score-weight interaction is controlled by parameter *β*.
Eqs (18) and (19). Refs. [65–67].	Behavioral noise *ϕ*_*i*_(*t*) with magnitude *η*.

Following a tradition in quantitative sociology [52, 54–61], we model agents via thresholds elements, i.e. we postulate that each agent *i* has an information potential *w*_*i*_(*t*) ≥ 0, and *i* activates whenever *w*_*i*_(*t*) is larger than a threshold *u*_*i*_ > 0:

$$\begin{array}{ccc} m_{i}\left(t\right) & = & \vartheta [w_{i}\left(t\right)-u_{i}],\;\;t=0,1,2,..., \end{array}$$ (1)

$$\begin{array}{ccc} w_{i}\left(t+1\right) & = & \left(1-m_{i}\left(t\right)\right)\sum_{j=1}^{N}q_{ij}\left(t\right)m_{j}\left(t\right), \end{array}$$ (2)

where *ϑ*(*x*) is the step function: *ϑ*(*x* < 0) = 0, *ϑ*(*x* ≥ 0) = 1. The factor (1 − *m*_*i*_(*t*)) in Eq (2) nullifies the potential after activation; hence an agent cannot be permanently active. The influence *q*_*ij*_(*t*)*m*_*j*_(*t*) of *j* on *i* is non-zero provided that *j* activates, *m*_*j*_(*t*) = 1. We assume that *q*_*ij*_ ≥ 0 and *q*_*ii*_ = 0, i.e. connections can only facilitate the potential generation. Given the freedom in choosing *q*_*ij*_, we take *u*_*i*_ = 1.

The continuous-time limit of Eqs (1) and (2) reduces to an integrate and fire model of neuronal dynamics [65–67].

In Eq (2), *q*_*ij*_(*t*) quantifies the influence of *j* on *i*. We parametrize it as

$$\begin{array}{c} q_{ij}\left(t\right)=q\tau_{ij}\left(t\right),\;\tau_{ii}\left(t\right)=0, \end{array}$$ (3)

$$\begin{array}{c} \sum_{j=1}^{N}\tau_{ij}\left(t\right)=1, \end{array}$$ (4)

where *q* is the maximal possible value of *q*_*ij*_(*t*). Now *τ*_*ij*_ is the weight of influence. Eq (4) reflects the fact that agents have limited attention [68–70]. This characteristics was also noted for neurons [71], and it is achieved via introducing non-normalized weights ${\tilde{\tau }}_{ij}\left(t\right)$ [71]:

$$\begin{array}{c} \tau_{ij}\left(t\right)={\tilde{\tau }}_{ij}\left(t\right)/\sum_{j=1}^{N}{\tilde{\tau }}_{ij}\left(t\right). \end{array}$$ (5)

Importantly, we do not pre-determine the network structure. The sum $q\sum_{j=1}^{N}\tau_{ij}\left(t\right)m_{j}\left(t\right)$ in Eq (2) is taken over all the agents, and the weights change in time as

$$\begin{array}{c} {\tilde{\tau }}_{ij}\left(t+1\right)=\tau_{ij}\left(t\right)+f\tau_{ij}^{\alpha }\left(t\right)m_{j}\left(t+1\right),\;\alpha >0, \end{array}$$ (6)

where a non-active *j* (*m*_*j*_(*t* + 1) = 0) does not change *τ*_*ij*_. In one version of the model *f* = const. Thus ${\tilde{\tau }}_{ij}\left(t\right)$ changes such that more active and more credible agents get more attention from neighbors. In Eq (6), $\tau_{ij}^{\alpha }\left(t\right)$ controls the extent to which *i* re-considers those links that did not attract its attention previously (conservatism): for *α*¬ ≈ 0 the weight with *τ*_*ij*_(*t*) ≈ 0 is not reconsidered in the next step. A similar structure was employed for modeling confirmation bias [72]. It also appears in a preferential selection model for network evolution [35].

Below we study the above model for *f* = const, and show that it leads to a non-trivial leadership scenario. To get richer scenarios, we shall introduce additional variables.

### Credibility scores

For each agent *i* we now introduce its credibility score *σ*_*i*_(*t*) ≥ 0, which is a definite feature of an agent at a given moment of time [73]. Credibility refers to the judgments made by a message recipient concerning the believability of a communicator. A more general definition of credibility (not employed here) should account for its subjective aspect; a message source may be thought highly credible by one perceiver and not at all credible by another [73].

Credibility scores interact with *m*_*i*_ and *τ*_*ij*_ by modulating the function *f* in Eq (6):

$$\begin{array}{c} f=f[\sigma_{j}\left(t\right)-\beta \sigma_{i}\left(t\right)],\;\beta =0,1, \end{array}$$ (7)

where we assume for simplicity

$$\begin{array}{c} f[x]=x\;\text{for}\;x>0;\;f[x]=0\;\text{for}\;x\leq 0. \end{array}$$ (8)

Thus for *β* = 1, the agent *i* reacts only on those with credibility score higher than *σ*_*i*_, whereas for *β* = 0 every agent *j* can influence *i* proportionally to its score *σ*_*j*_. For convenience, we restricted *β* = 0, 1 to two values. (Note that if we define *f*[*x*] to be a positive constant for *x* ≤ 0, then the situation without scores can be described via *β* → ∞.)

Dynamics of *σ*_*i*_ is determined by the number of agents that follow *i* and by the amount of attention those followers pay to the messages of *i*:

$$\begin{array}{c} \sigma_{i}\left(t+1\right)=\left(1-\xi_{1}\right)\sigma_{i}\left(t\right)+\xi_{2}\sum_{k=1}^{N}m_{i}\left(t\right)\tau_{ki}\left(t\right), \end{array}$$ (9)

where *ξ*_1_ and *ξ*_2_ are constants: 1 ≥ *ξ*_1_ > 0 quantifies the credibility score loss (forgetting), while the term with *ξ*_2_ > 0 means that every time the agent *i* activates, its score increases proportional to the weight *τ*_*ki*_(*t*) of its influence on *k*. If *i* is not active, *m*_*i*_(*t*) = 0, its score decays.

Development of complex network theory motivated many models, where the links and nodes are coupled [46, 47, 74–81]; see [82] for an extensive review. Neurophysiological motivation for studying such models comes from the synaptic plasticity of neuronal connections that can change on various time scales [65, 83].

The ingredients in the evolution of the credibility score Eq (9) do resemble the notion of fitness, as introduced for models of competing animals [38]. There the fitness determines the probability of winning a competition. Similar ideas were employed for modeling social diversity [39]; see [40] for a review.

### Initial conditions

All agents are equivalent initially:

$$\begin{array}{c} \sigma_{i}\left(0\right)=0. \end{array}$$ (10)

The initial network structure is random [cf. Eq (5)]

$$\begin{array}{c} \tau_{ij}\left(0\right)=n_{ij}/\sum_{k=1}^{N}n_{ik},\;n_{ij}\in \left[0,b\right],\;n_{ii}=0, \end{array}$$ (11)

where *n*_*ij*_ are independent random variables homogeneously distributed over the interval [0, *b*]. Now

$$\begin{array}{c} \phi_{i}\equiv \sum_{k=1}^{N}\tau_{ki}\left(0\right), \end{array}$$ (12)

measures the initial cumulative influence of *i* (i.e. its initial social capital and estimates the initial rate of score generation; cf. Eq (9). (Financial capital is money. Physical capital is tools, machinery *etc*. Human capital is people. Social capital is the relationship among persons. Human capital resides in people; social capital resides in the relations among them [1].)

For *m*_*i*_(0) we impose initial conditions, where some agents are activated initially (by a news or discussion subject), i.e. *m*_*i*_(0) are independent random variables:

$$\begin{array}{c} \text{Pr}[m(0)=1]=\gamma ,\;\text{Pr}[m(0)=0]=1-\gamma ,\;\gamma \leq 1/2. \end{array}$$ (13)

### Thresholds of collective activity

The only way the initial activity can be sustained is if the agents stimulate each other (as it happens in a real discussion process). Our numerical results show that with initial conditions Eq (13) there exists a sustained activity regime. Specifically, there are two thresholds $Q^{+}$ and $Q^{-}$, so that for $q\geq Q^{+}$ the initial activity is sustained indefinitely,

$$\begin{array}{c} \sum_{i=1}^{N}m_{i}\left(t\right)>0,\;\text{if}\;q\geq Q^{+}. \end{array}$$ (14)

If $q\leq Q^{-}$ the initial activity decays in a finite time *t*_0_ (normally few time-steps)

$$\begin{array}{c} \sum_{i=1}^{N}m_{i}\left(t\geq t_{0}\right)=0,\;\text{if}\;q\leq Q^{-}. \end{array}$$ (15)

For $Q^{+}>q>Q^{-}$ the activity sustaining depends on the realization of random initial conditions *m*_*i*_(0) and *τ*_*ij*_(0): either it is sustained indefinitely or it decays after few steps. Qualitatively, the activity is sustained if sufficiently well-connected agents are among the initially activated ones; see below. $Q^{+}$ and $Q^{-}$ depend on all the involved parameters, their numerical estimates are given below in Eq (17). Note that $Q^{-}>1$, since for *q* < 1 no activity spreading is possible: even the maximal weight *τ*_*ik*_ = 1 cannot activate *i*; see Eqs (2) and (3).

Emergent networks (described below) depend mainly on parameters *α* and *β*; see Table 1. Other parameters can be important for supporting activity, i.e. $Q^{+}$ and $Q^{-}$ depend on them, but are not crucial for determining the type of the emerging network. So we fix for convenience [cf. Eqs (13), (11) and (9)]:

$$\begin{array}{c} N=100,\;\gamma =0.5,\;b=10,\;\xi_{1}=0.1,\;\xi_{2}=1. \end{array}$$ (16)

For these parameters and (for example) *α* = *β* = 1, we got numerically

$$\begin{array}{c} Q^{-}=2.19,\;Q^{+}=2.65. \end{array}$$ (17)

### Behavioral noise

The deterministic firing rule Eq (1) can be modified to account for agents with behavioral noise. We want to make it possible for an agent to activate (not to activate) even for sub-threshold (super-threshold) values of the potential. The noise will be implemented by assuming that the threshold *u*_*i*_ + *v*_*i*_(*t*) has (besides the deterministic component *u*_*i*_ discussed in Eq (1)) a random component *v*_*i*_(*t*). These quantities are independently distributed over *t* and *i*. Now *v*_*i*_(*t*) is a trichotomic random variable, which takes values *v*_*i*_(*t*) = ±*V* with probabilities $\frac{\eta }{2}$ each, and *v*_*i*_(*t*) = 0 (no noise) with probability 1 − *η*. Hence *η* describes the magnitude of the noise. We assume that *V* is a large number, so that with probability *η*, the agent ignores *w*_*i*_ and activates (or does not activate) randomly. Thus, instead of Eq (1), we now have

$$\begin{array}{c} m_{i}\left(t\right)=\vartheta \left[\phi_{i}\left(t\right)\left(w_{i}\left(t\right)-u_{i}\right)\right], \end{array}$$ (18)

$$\begin{array}{c} \text{Pr}\left[\phi_{i}\left(t\right)=1\right]=1-\eta ,\;\text{Pr}\left[\phi_{i}\left(t\right)=-1\right]=\eta , \end{array}$$ (19)

where *ϕ*_*i*_(*t*) are independent (over *i* and over *t*) random variables that equal ±1 with probabilities Pr[*ϕ*_*i*_ = ±1].

Qualitatively the same predictions are obtained under a more traditional (for the neuronal network literature [65]) model of noise, where the step function in Eq (1) is replaced by a sigmoid function.

## Laissez-faire leadership

**Fig 1:**
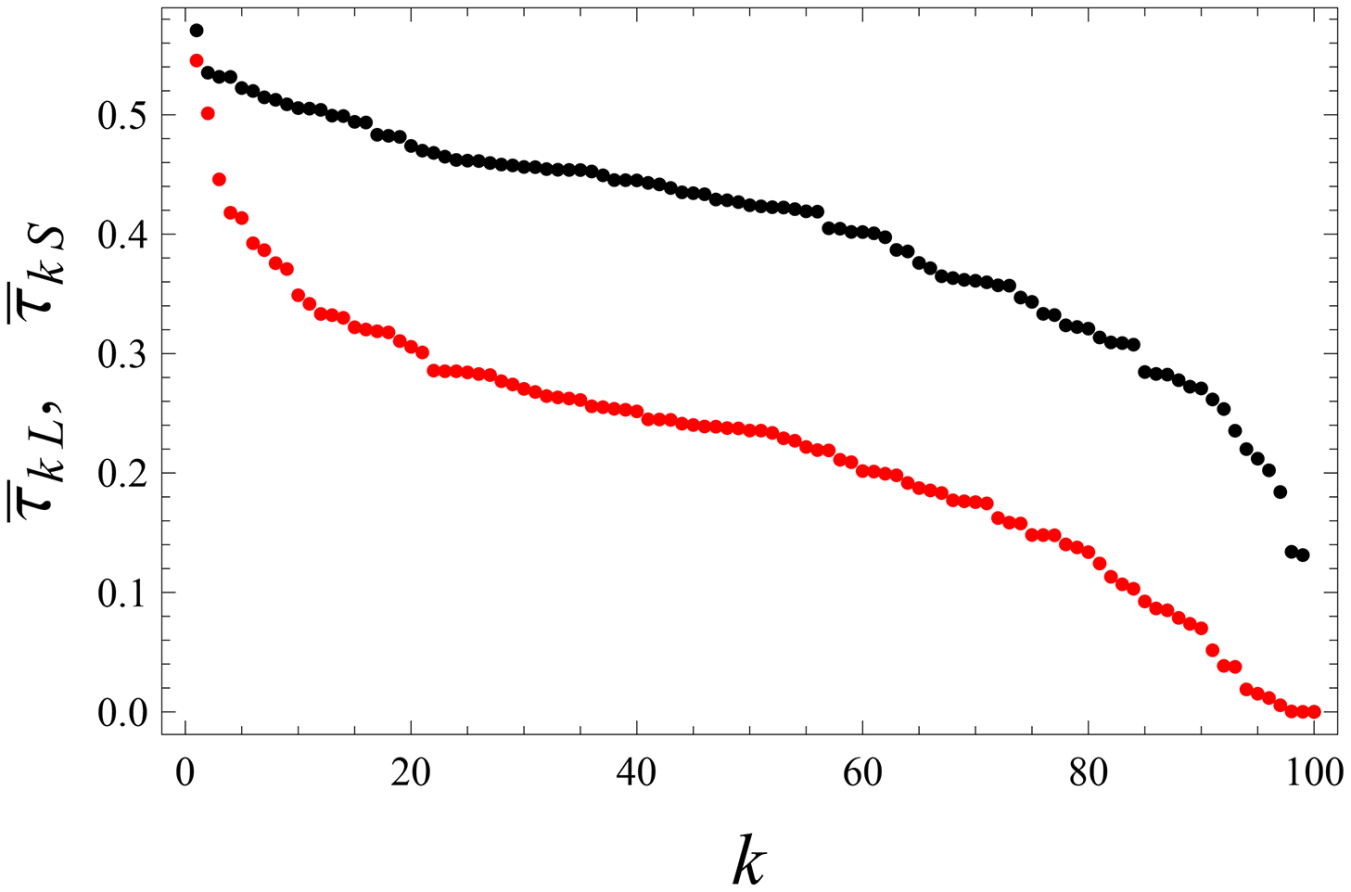
Laissez-faire leadership. In Eq (6) we set *f* = 1 and *α* = 1. The behavioral noise is weak: *η* = 0.07; cf. Eqs (18) and (19). The intergent coupling *q* = 2 is sizable, but is smaller than Eq (17). The observation time: *T* = 600; see Eq (20). Black points (upper curve): the average weights ${\bar{\tau }}_{kL}$ by which the leader *L* influences other agents; see Eq (20). Red points (lower curve): ${\bar{\tau }}_{kS}$ that quantify the influence of the leading sub-leader *S* on other agents. Here ${\bar{\tau }}_{kS}$ and ${\bar{\tau }}_{kL}$ were separately arranged in the decreasing order over *k*.

**Fig 2:**
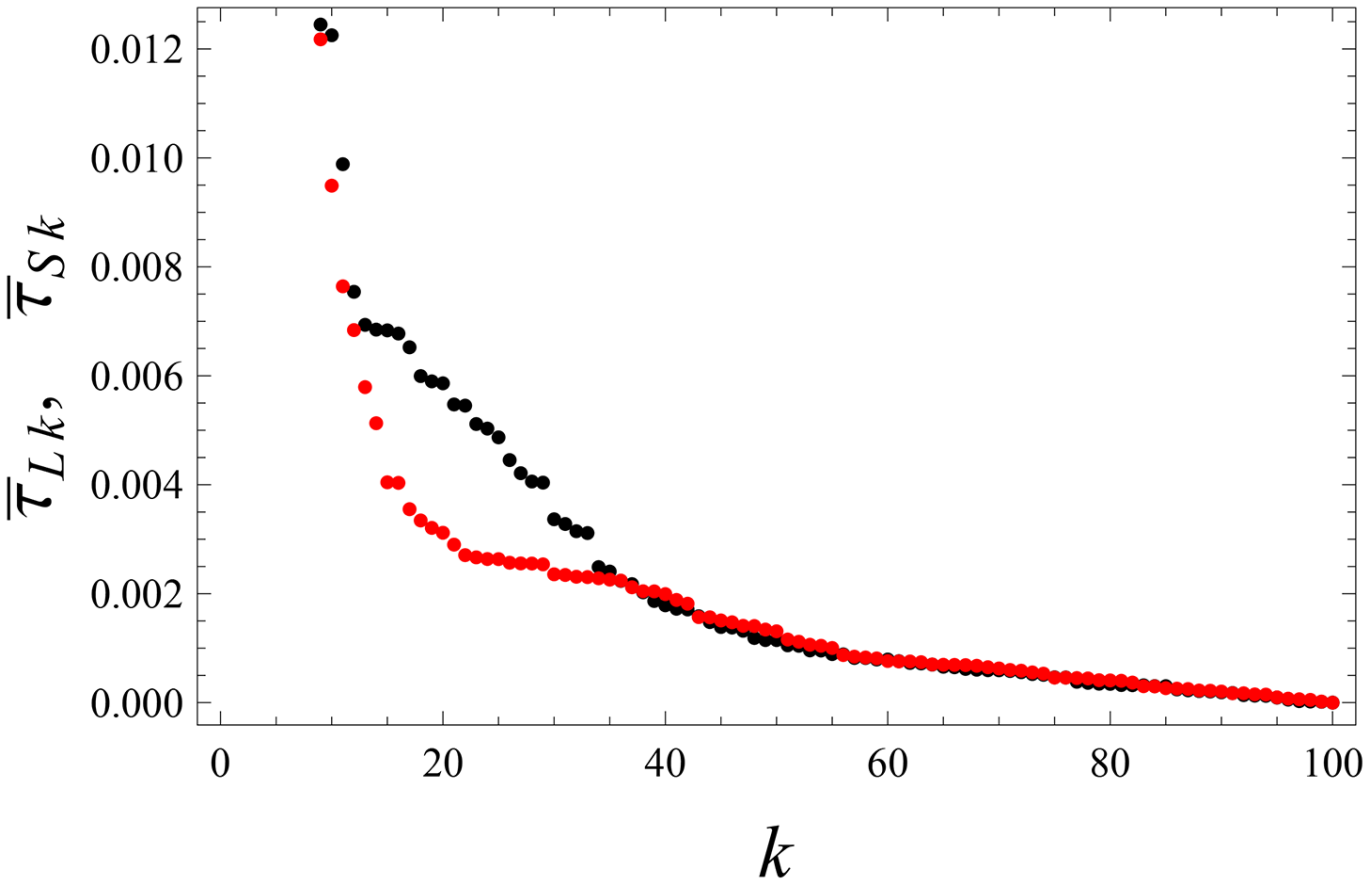
Laissez-faire leadership. The same parameters as in Fig 1, but for the weights ${\bar{\tau }}_{Lk}$ and ${\bar{\tau }}_{Sk}$ that quantify the influence of followers on the leader *L*, and on the sub-leader *S*, respectively. Again, ${\bar{\tau }}_{Sk}$ and ${\bar{\tau }}_{Lk}$ were separately arranged in decreasing order. Note that the influence of *L* and on *S*, and *S* on *L* are comparable: ${\bar{\tau }}_{SL}=0.545378$ and ${\bar{\tau }}_{LS}=0.535170$.

**Fig 3:**
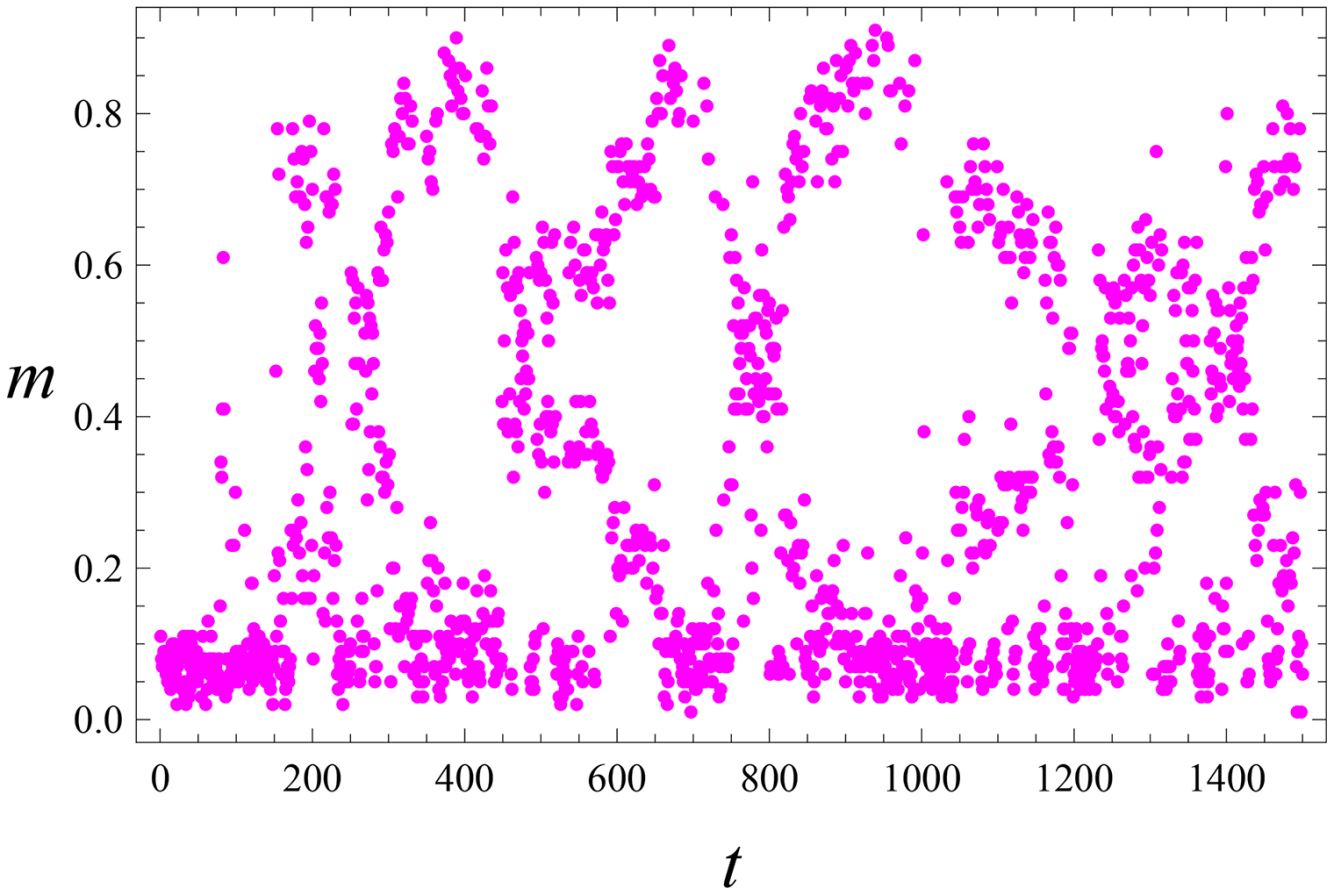
Laissez-faire leadership. The same parameters as in Fig 1. Collective activity $m\left(t\right)=\sum_{i=1}^{N}m_{i}\left(t\right)$ versus time *t*. It is seen that *m*(*t*) displays irregular (noisy) behavior.

**Fig 4:**
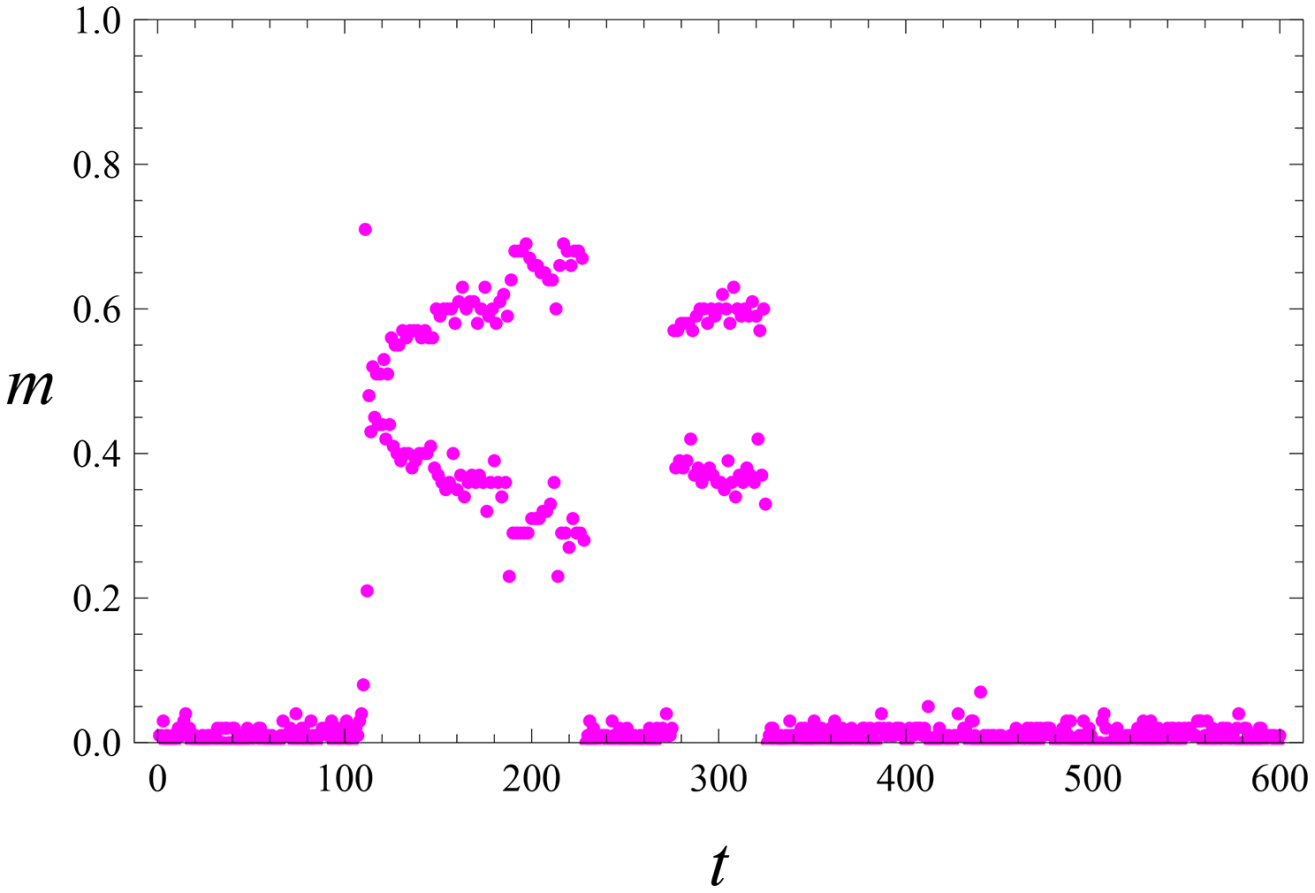
Laissez-faire leadership. The same parameters as in Figs 1 and 3 Collective activity $m\left(t\right)=\sum_{i=1}^{N}m_{i}\left(t\right)$ versus time *t*, but for a weaker noise with magnitude *η* = 0.01.

We shall study Eqs (1) and (2) under a weak-noise (*η* ≪ 1 in Eqs (18) and (19)), but without scores, i.e. we take *f*[*x*] = const in Eq (6). The magnitude *q* of the inter-agent interaction (see Eq (3)) is taken so that no activity is sustained without the noise, i.e. $q<Q^{-}$; cf. Eqs (15) and (17). Hence we are looking for a regime, where both noise and inter-agent interactions are essential; see Figs 1–4. The activity of the system—as measured by $m\left(t\right)=\frac{1}{N}\sum_{i=1}^{N}m_{i}\left(t\right)$—is now much larger than the noise magnitude *η*, i.e. the noise is amplified; see Fig 3. We shall see that in this regime there does emerge a laissez-faire leadership scenario.

In this noisy situation, the network structure is studied via time-averaged weights:

$$\begin{array}{c} {\bar{\tau }}_{ij}=\frac{1}{T}\sum_{t=s}^{T+s}\tau_{ij}\left(t\right), \end{array}$$ (20)

where the observation time *T* is sufficiently large.

The average, cumulative influence of an agent *k* to all other agents is quantified by $\sum_{i=1}^{n}{\bar{\tau }}_{ik}$. The leader can be defined by the maximum of this quantity over *k*. We saw that there emerges a leader (*L*) that collects feedback from its many other agents (followers); see Figs 1 and 2. There is also a sub-leader *S* (or few sub-leaders depending on the realization of the noise and the initial state) for which $\sum_{i=1}^{n}{\bar{\tau }}_{ik}$ is next to maximal over *k*. The sub-leader *S* has its own followers and does collect feedback from them. The influence of *L* and *S* on other agents is larger than the back-influence of those agents; see Figs 1 and 2. Many followers are shared between *L* and *S*. All followers influence each other, but with much smaller weights $O(\frac{1}{N})$.

The relation between *L* and *S* is not hierarchical, since they drive each other with comparable magnitudes; see Figs 1 and 2. They are distinguished from each other by the fact that *L* has more followers and influences them stronger.

The collective activity $m\left(t\right)=\frac{1}{N}\sum_{i=1}^{N}m_{i}\left(t\right)$ shows irregular (chaotic) oscillations; see Fig 3. Generally, the emergence of *L* and *S* takes few such oscillations, i.e. a rather long time (*t* ∼ 500 for parameters of Figs 1–3). For even smaller magnitude of noise, the system activates via relatively rare bursts; see Fig 4.

This leadership is not an epiphenomenon. Indeed, we can intervene into the system and block the activity of *L* and *S* by suddenly raising their activation thresholds from *u* = 1 to some very large value. As compared to the same situation, but without intervention, the time-averaged overall activity

$$\begin{array}{c} \frac{1}{TN}\sum_{t=s}^{T+s}\sum_{i=1}^{N}m_{i}\left(t\right) \end{array}$$ (21)

does decrease by 20–30%, and is recovered only after a long time, when new leader and sub-leader emerge. (No statistically significant activity reduction was seen when the same amount of non-leaders was blocked). For the situation shown in Figs 1–4, the intervention was realized by letting the system to evolve for *t* = 1, …, 600, suddenly blocking *L* and *S*, taking *s* = *T* = 600 and then looking at the activity change (reduction) by calculating Eq (21) with and without intervention.

Note that the scenario is only weakly dependent on the value of *α*, but it is essentially based on the noise. For the noise-free situation *η* = 0 [see Eqs (18) and (19)], we get either no activity whatsoever (for a sufficiently small *q*), or a no-leader activity-sustaining for a larger *q*. Another essential aspect is the sufficiently fast adaptation, as quantified by the value of *f*[*x*] = const in Eq (6). If for parameters of Figs 1–4 we take *f* = 0.25 (instead of *f* = 1), no activity above the noise magnitude *η* is detected.

This scenario is similar to the laissez-faire leadership (as discussed e.g. in [1, 2, 12]): substantially autonomous followers, the existence of sub-leaders, no hierarchy between the leader and sub-leader(s), irregular activity structure. And yet our model shows that this is a real leadership: in contrast to what people sometimes think and say about laissez-faire leaders, our model shows that when blocking the leader and sub-leader the activity of the system does decrease. Moreover, it takes a long time to establish a laissez-faire leader and to replace it. We stress however that the effect of the laissez-faire leadership is visible for sufficiently long times and hence requires time-averaged indicators.

Thus the present model shows that the laissez-faire leadership scenario emerges in a noisy (i.e. information rich) situation, where the inter-agent interaction is sizable, but it not so strong that the activity is sustained without noise.

## Participative leadership

### Single participative leader

We solve dynamics with credibility scores Eqs (1), (2) and (6)–(9) for $q>Q^{-}$; hence there is a possibility for the sustaining activity. Noise is omitted, *η* = 0 in Eqs (18) and (19), because it is irrelevant; see below. The parameters in Eqs (6) and (7) are

$$\begin{array}{c} \alpha =\beta =1. \end{array}$$ (22)

**Fig 5:**
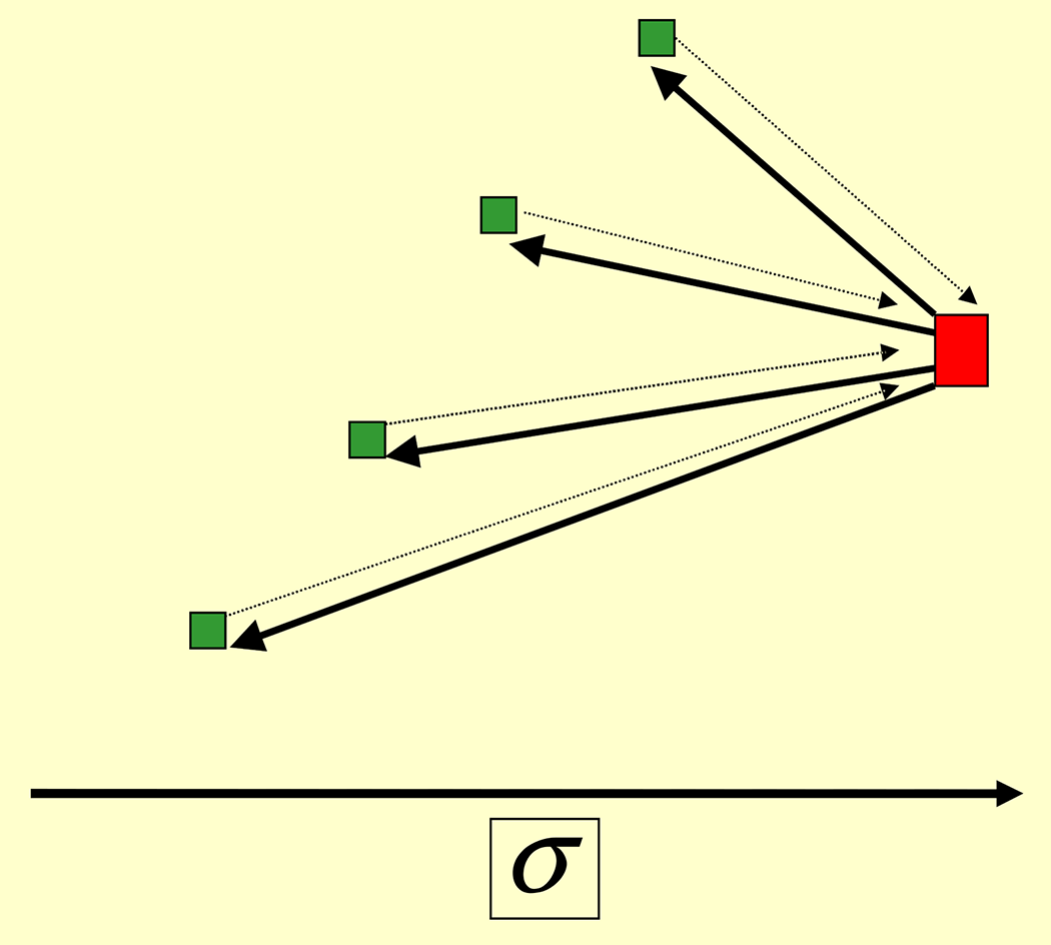
Participative leadership. Emergent network structures according to Eqs Eqs (1)–(11) and (13). Parameters are chosen from Eqs (16) and (17) and $q>Q^{-}$. *Single Participative leader*: *α* = *β* = 1; see Eqs (6) and (7). The leader *L* (red square) has the highest score (≃500) and stimulates all other agents (followers, green squares) with the maximal weight *τ*_*i*L_ = 1 (bold arrows). Followers (green squares) have different credibilities $\sigma_{i}=O\left(1/N\right)$; each of them stimulates the leader with weights $\tau_{Li}=O\left(1/N\right)$: a follower with a larger score influences the reader stronger.

After ≃20 time-steps the system enters into a stationary state, where the activation frequencies do not depend on time. There emerges a leader (*L*) that drives all the other agents (followers) with the maximal weight *τ*_*kL*_ = 1 [cf. Eqs (1) and (2)], i.e. each follower is influenced only by *L*; see Fig 5 for schematic representation. Hence *L* emergence in a much shorter time than the laissez-faire leader, and *L* also influences the followers much strongly.

Credibility scores *σ*_*k*_ of followers and their influence weights *τ*_*Lk*_ reach quasi-stationary values over a larger time [≃100 for parameters of Eq (16)]. *L* has the largest final score (*σ*_*L*_ ≫ 1). Followers have much lower scores and influence *L* via smaller weights $\tau_{Lk}=O\left(1/N\right)<1$. Followers do not influence each other (in contrast to the laissez-faire scenario) precisely because the influence of *L* on its follower is maximal; see Eq (4). The activity of the leader (hence the overall activity) is sustained due to cumulative feedback from followers to *L*. Hence this is a participative leadership scenario.

**Fig 6:**
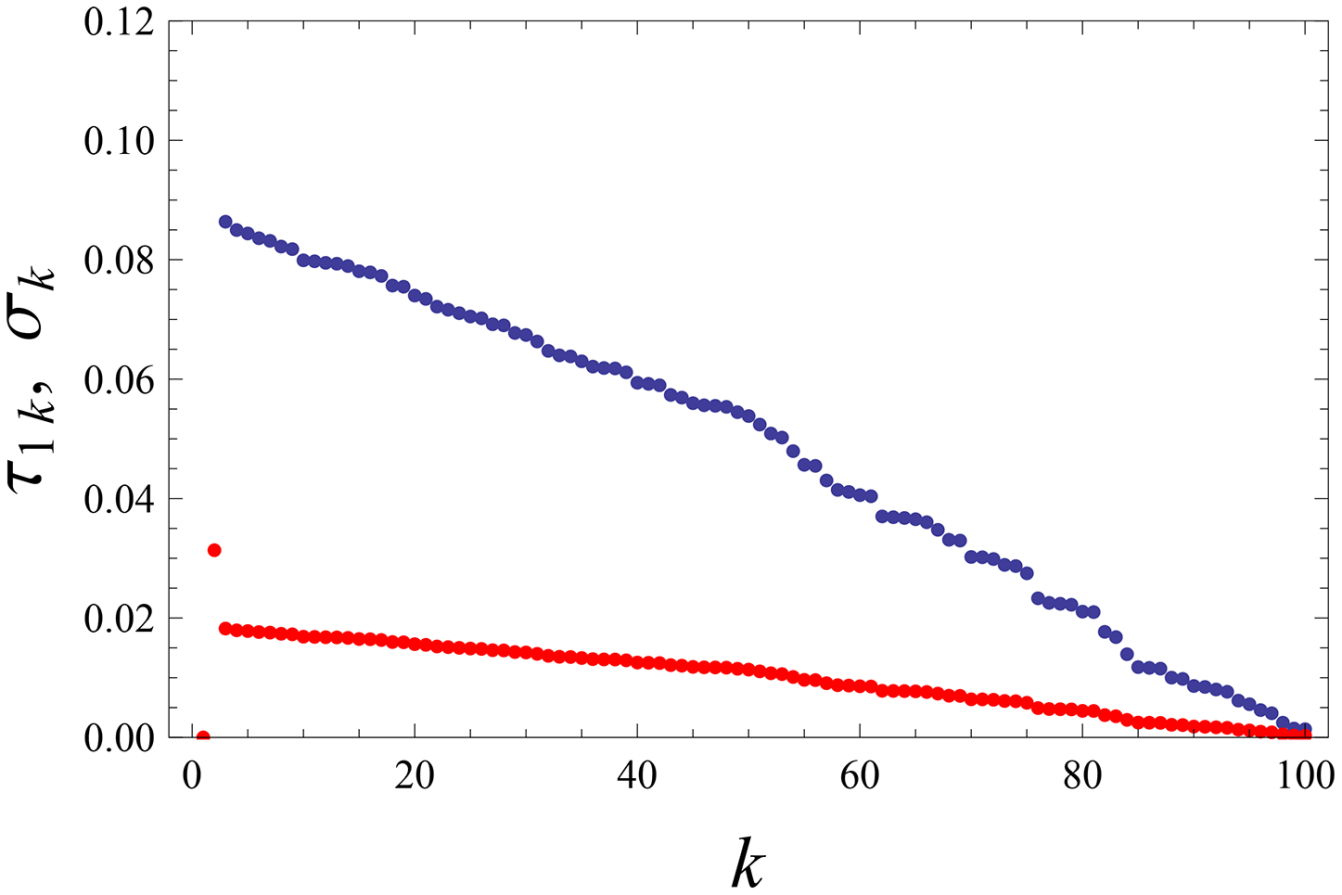
Participative leadership: single emergent leader. Distribution of stationary credibilities *σ*_*k*_ (blue points, upper curve) and weights *τ*_1*k*_ (red points, lower curve) by which the agent with rank *k* (*N* ≥ *k* ≥ 2) influences the leader (*k* = 1). The agents are ranked according to their final score: *k* = 1 is the highest-score agent (leader), *k* = *N* is the lowest score agent. Eqs (1)–(9) and (13) are solved for Eq (16) and *q* = 2.5. The dynamics was followed by 200 time-units.

**Fig 7:**
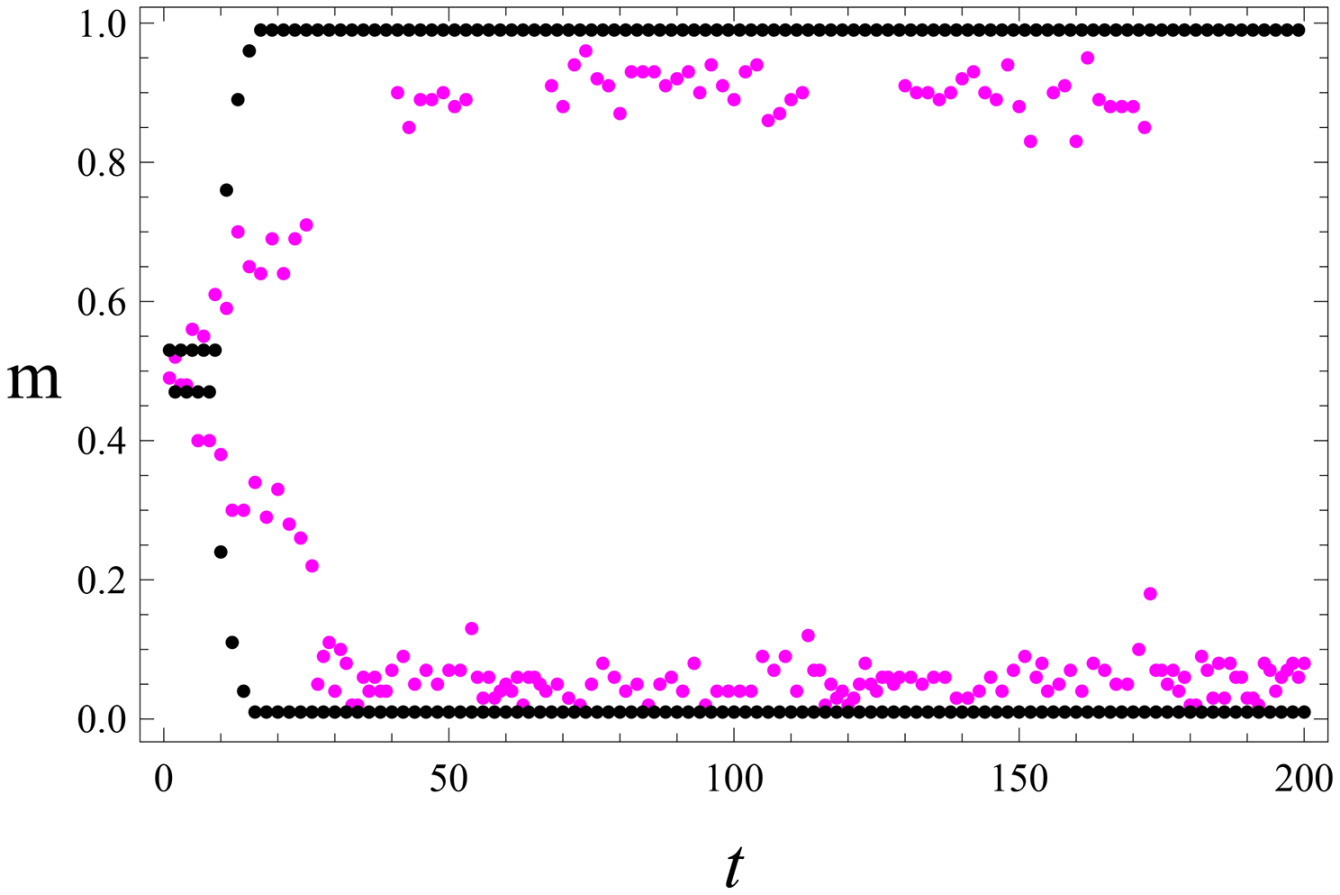
Participative leadership. Single emergent leader. The collective activity $m\left(t\right)=\frac{1}{N}\sum_{k=1}^{N}m_{k}\left(t\right)$ as a function of time *t* for *q* = 2.7 (other parameters are the same as in Fig 6). Black points (two straight lines): the noiseless situation *η* = 0. Magenta points: *η* = 0.05; cf. Eqs (18) and (19). In the noiseless situation *m*(*t*) takes only two values 0.01 (the leader is active) and 0.99 (followers are active). For the noisy situation *m*(*t*) assumes two well-separated sets of values at ∼0.1 and ∼0.9, respectively.

The score distribution of followers is such that a follower with a higher score influences the leader more; see Fig 6. In the noiseless situation the dynamics is strictly synchronized: only *L* fires in one time-unit. In the next time-unit *L* is passive, while followers activate together. The strict synchronization disappears after introducing a small noise; see Eqs (18) and (19) and Fig 7, where the collective activity $m\left(t\right)=\frac{1}{N}\sum_{k=1}^{N}m_{k}\left(t\right)$ is monitored with and without noise. Other characteristics of the scenario stay intact.

Initially, *L* had to be among activated agents: *m*_*L*_(0) = 1, *and* it was most probably having the largest social capital [in the sense of Eq (12)] among the initially activated agents; this is the case in ≃95% of (random) initial conditions. Hence the leader emerges due to the amplification of its initially small advantages over other agents.

Comparing the laissez-faire leader with the present one, we see that introducing credibility scores enforces a hierarchy, i.e. it eliminates connections between the followers and maximizes the influence of the leader.

### Externally imposed versus emergent leadership

It is of clear importance to understand when and whether externally imposed leaders can compete with those that emerged from within the group [1]. We model imposed leaders by externally driving (sponsoring) the activity of certain agent(s). To this end, we add a term (1 − *m*_*i*_(*t*))*r*_*i*_ to the right-hand-side of Eq (2), where *r*_*i*_ ≥ 0 is the rate of potential generation that does not depend on inter-agent interaction. In particular, *r*_*i*_ can be an externally imposed rate. Thus Eq (2) becomes

$$\begin{array}{c} w_{i}\left(t+1\right)=\left(1-m_{i}\left(t\right)\right)\sum_{j=1}^{N}q_{ij}\left(t\right)m_{j}\left(t\right)+\left(1-m_{i}\left(t\right)\right)r_{i}. \end{array}$$ (23)

We focus on the situation, where only one agent is externally driven:

$$\begin{array}{c} r_{1}>1,\;r_{i\geq 2}=0, \end{array}$$ (24)

i.e. the first agent activates with the maximal frequency 0.5; cf. Eqs (1) and (2).

Now for $q<Q^{-}$ the first agent becomes a leader. For $Q^{-}<q<Q^{+}$ (and Eq (22)) it can—depending on initial conditions—become a participative leader, and it generally does not become a leader for $q>Q^{+}$: the emergent leader takes over the externally driven agent.

### Hierarchy of leaders

**Fig 8:**
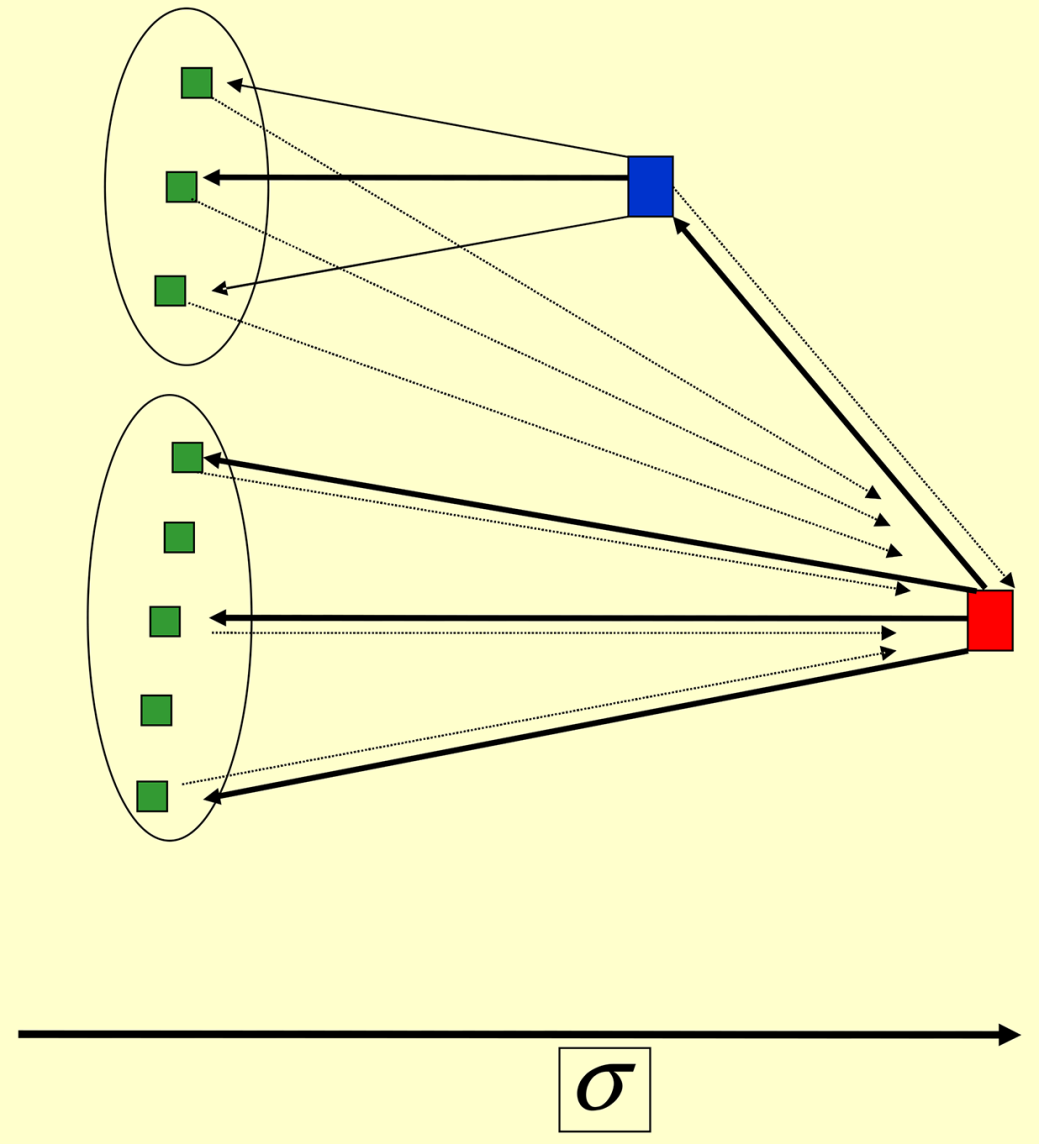
Participative leadership. *Hierarchic leadership*: *α* = 2, *β* = 1. (Other parameters are the same as in Fig 5.) The followers are divided into two groups, strongly driven by respectively leader (red) and sub-leader (blue). The feedback is collected by the leader only.

If instead of Eq (22) we employ 1.5≲α and *β* = 1, we get a hierarchic leadership scenario; see Fig 8. There emerge one leader and few sub-leaders. The precise number of sub-leaders depends on initial conditions. Each sub-leader has followers that are not directly influenced by the leader. The latter influences only their sub-leader; see Fig 8. In a sense the leader delegates some of its influence to the sub-leader(s). But the main feature of the participative leadership is kept: only the top leader collects feedback from all other agents.

Note the difference with sub-leaders within the laissez-faire scenario: there ${\bar{\tau }}_{LS}≃{\bar{\tau }}_{SL}$, i.e. the leader and sub-leader influence each other with comparable weights. In contrast here *τ*_*LS*_ ≪ *τ*_*SL*_ = 1, i.e. the influence of the leader is maximal and is much larger than the influence of the sub-leader on the leader. Thus the participative leadership is hierarchical, in contrast to the laissez-faire scenario. This hierarchy is introduced by credibility scores that were absent in the latter scenario.

## Autocratic leadership

### Single autocratic leader

We turn to studying Eqs (1), (2) and (6)–(9) with

$$\begin{array}{c} \alpha =1,\;\beta =0, \end{array}$$ (25)

and for $q>Q^{-}$, i.e., for initial conditions where the activity can be sustained. We get $Q^{-}=2.18$ under Eqs (25) and (16); cf. Eq (17).

**Fig 9:**
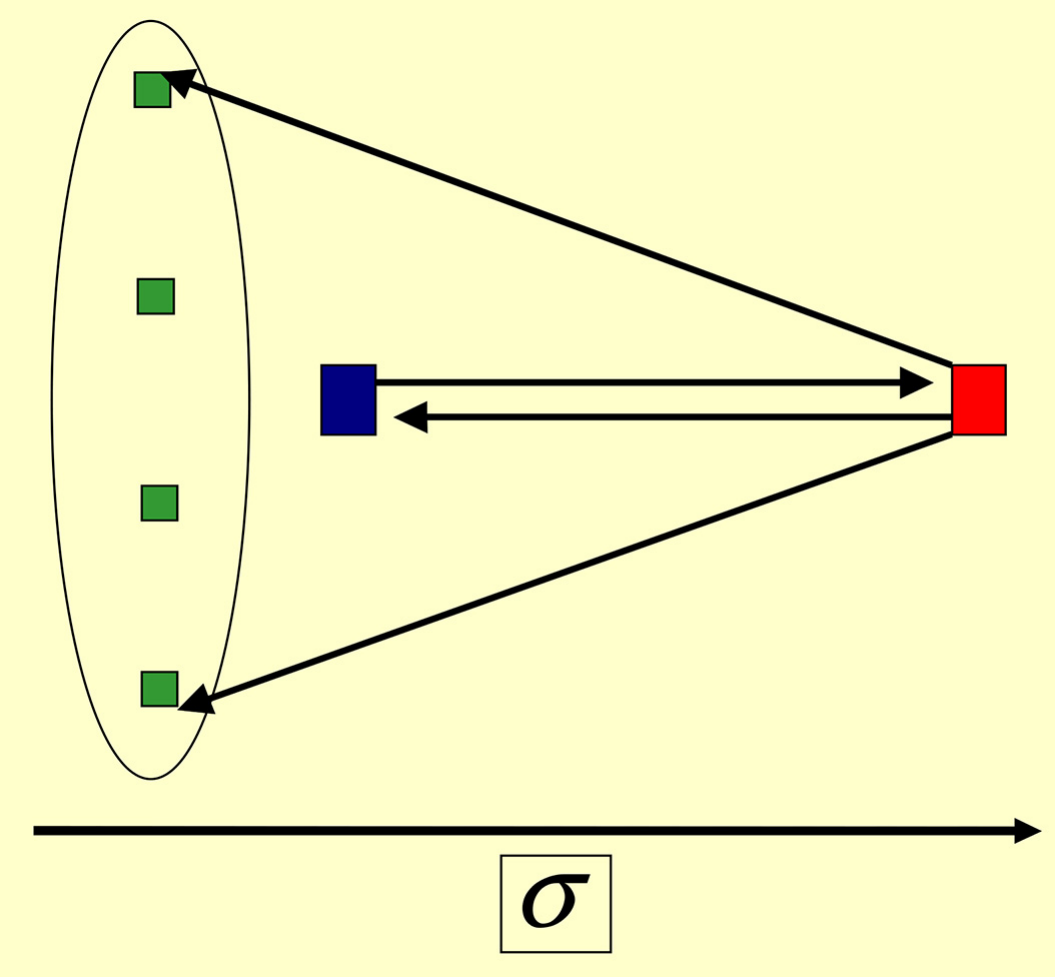
Autocratic leadership. *Single autocratic leader*: *α* = 1, *β* = 0 and $q>Q^{-}$; see Eqs (6), (7), (14) and (15). Other parameters are chosen according to Eq (16). The leader (red square) stimulates others (green squares) and is stimulated by the helper (blue square). All these stimulations have the maximal weight equal to 1. All other agents are passive spectators with zero score.

**Fig 10:**
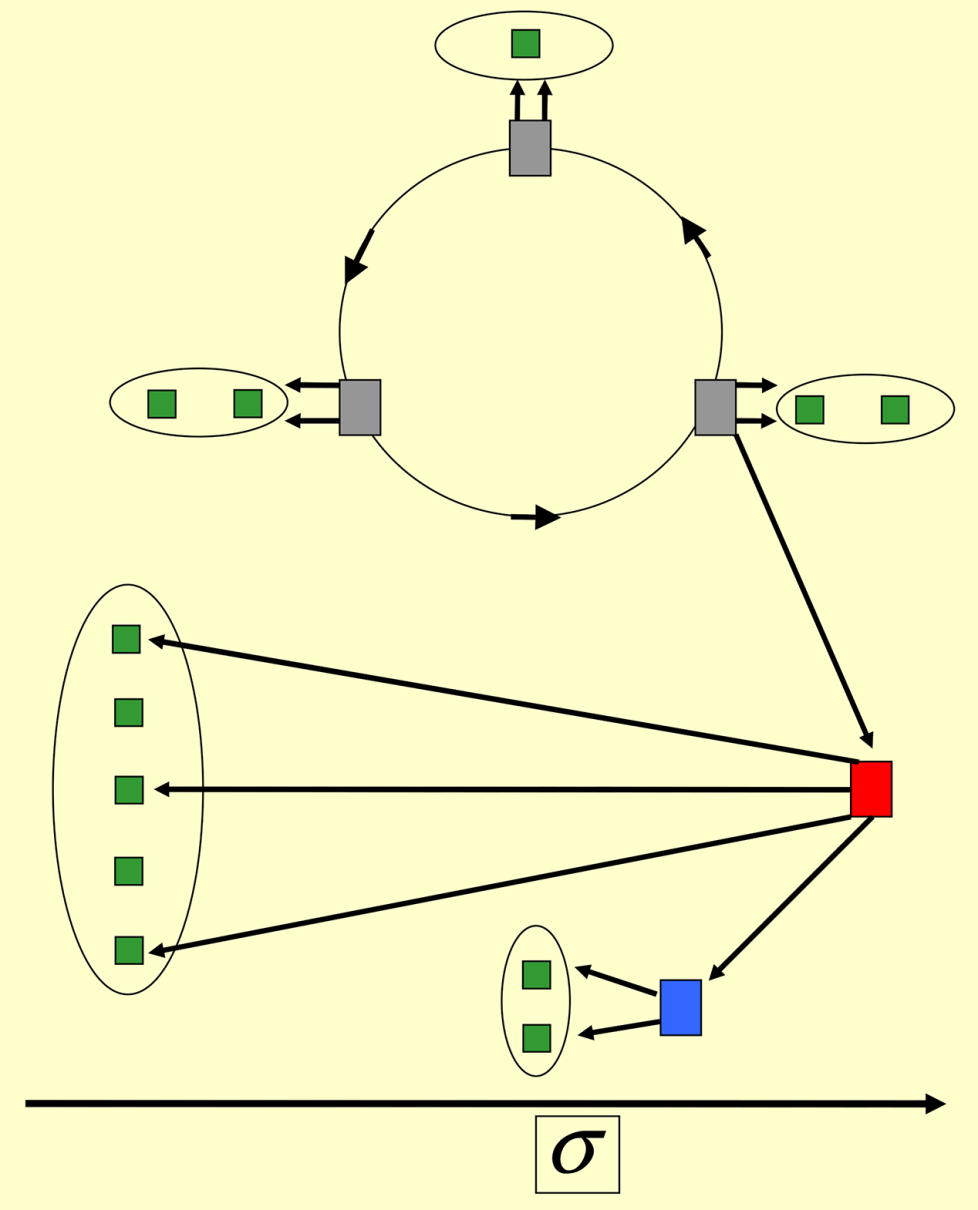
Autocratic leadership. *Hidden leadership*: *α* = 1.5, *β* = 0 (other parameters are those of Fig 9). The highest-score agent (red square) is driven by a circle of agents that drive each other cyclically (grey squares). The official leader (red square) has the largest number of followers (green squares), though each gray agent can have its own followers. All followers are driven by the maximal weight, have neglegible credibilities and do not feedback.

In ≃20 time-steps, there emerges a leader *L* that has the highest score and that influences all other agents with the maximal weight *τ*_*kL*_ = 1; see Figs 9 and 10. However, it gets feedback only from one agent *H* that emerges together with *L*, and that stimulates *L* with the maximal weight, *τ*_LH_ = 1. Hence, *H* connects to no other agent. The only role of *H* is that it stimulates the leader; see Figs 9 and 10. The long-time score of *H* is next to the largest, e.g., for *q* = 2.5 and Eq (16) we get *σ*_*L*_ ≃ 500, *σ*_*H*_ ≃ 5, while *σ*_*k*_ ≃ 0 for *k* ≠ *L*, *H*.

For all other agents *k* (*k* ≠ *L*, *H*), the influence on *L*, on *H*, and on each other (i.e., the magnitude of *τ*_*ik*_ for *k* ≠ *L* and *k* ≠ *H*) is negligible; they are completely passive followers. We have here an autocratic leadership scenario, since no feedback is present from the followers to the leader; see Figs 9 and 10.

A pertinent question is whether the autocratic *L* had to have (initially) a large social capital; cf. Eq (12). The answer is definitely no. In ≃50% of initial conditions *L* did not have a large social capital initially, although both *L* and *H* have to be among the initially active agents *m*_*L*_(0) = *m*_*H*_(0) = 1; cf. Eqs (13) and (16). In contrast to the participative scenario, the selection of the autocratic leader is not fully determined by its social capital.

Another difference with respect to the participative scenario is that now *Q*^+^ = ∞. For whatever the large value of *q*, there are initial states where the activity is not sustained; in particular, it ceases before any leader emerges. In this sense the autocratic leadership is less stable. (However, the scenario is stable with respect to introducing the noise Eqs (18) and (19)). To illustrate this point, note that the activity is not sustained—and hence no definite network structure emerges—for regular initial conditions *τ*_*ij*_(0) = 1/(*n* − 1) (instead of Eq (11)). For the participative situation this homogeneous initial network structure only delayed (by an order of magnitude) the convergence towards the stationary structure. Thus, the emergence of the autocratic leader (and its helper) demands initial inhomogeneity of the network.

Another implication of *Q*^+^ = ∞ is that the externally driven agent (cf. Eq (24)) does always have a chance to become an autocratic leader.

### Hidden leadership

We continue to focus on *β* = 0, but now 1.5≲α; cf. Table 2. Instead of a single helper *H*, we get (depending on the initial conditions) a few agents *H*_1_, *H*_2_, …; see Fig 10. The agents act on each other *cyclically*, and although they have lower credibility scores than *L*, the one with the highest score (*H*_1_) drives *L* with the maximal weight *τ*_*LH*_1__ = 1. Thus *H*_1_, *H*_2_, … are *hidden and real* leaders. They are hidden because their score is not large; it is smaller than the score of *L*, but it is real because they influence each other strongly, and one of them influences *L*, which has the maximal score. Importantly, the influence of *H*_1_ on *L* is one-way; *H*_1_ does not get any feedback from *L*, in contrast to the previous scenario with the single helper. Thus, within the group *H*_1_, *H*_2_, … the score is not important, since these agents drive each other cyclically. However, *L* is still driven by the highest-score (among *H*_1_, *H*_2_, …), agent *H*_1_.

**Table 2:** Leadership scenarios for different parameters of Eq (7) and *f*[*x*] given by Eq (8). For intermediate values of the parameters (e.g. 1 < *α* < 1.5) we get mixtures of the corresponding scenarios, or one of the scenarios is selected depending on initial conditions.

	α≳1.5	*α* ≃ 1	α≲0.5
*β* = 1	hierarchy (participative)	single leader (participative)	succession of leaders (autocratic)
*β* = 0	hidden leaders (autocratic)	leader + helper (autocratic)	succession of leaders (autocratic)

Three further details can be present in this scenario, depending on initial conditions. First, it is also possible that *L* belongs to the group of hidden leaders *H*_1_, *H*_2_, …. Second, the scenario may be accompanied by a hierarchic structure, where the leader influences a sub-leader that drives its own group of followers; see Fig 10. Third, some of *H*_*k*_ (most frequently *H*_1_) can have their own followers, that are not the followers of *L*. Still, *L* has the largest number of followers and thereby the largest credibility score; see Fig 10.

### Coalition of autocratic leaders (duumvirate)

**Fig 11:**
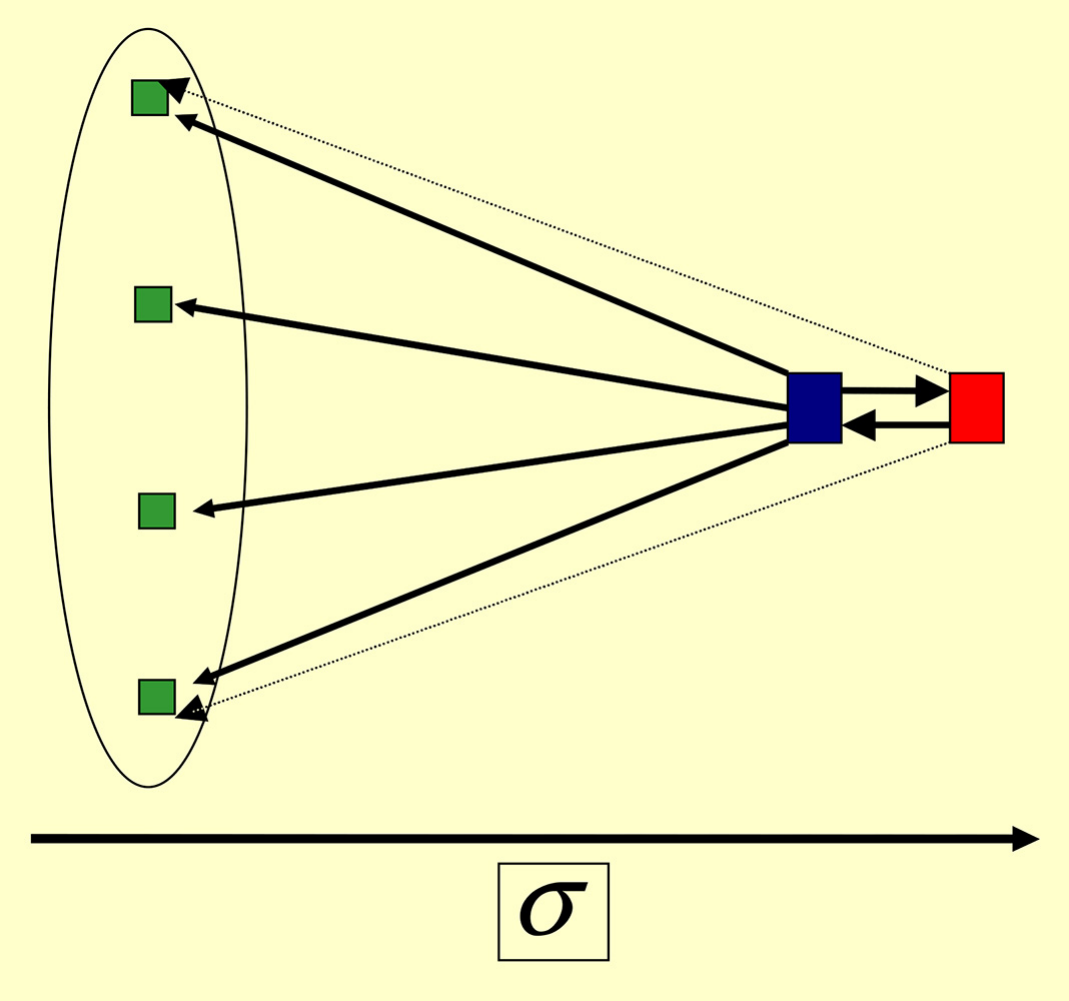
*Coalition* of two leaders. Here *α* = 0.25, *β* = 1 and Eq (16). The red (blue) agent has the highest (one but highest) score. However, the real leader is now the blue agent, since it stimulates all other agents with the weight equal to 1. The red agent stimulates the blue one with the weight 1, and all other agents with the weight ≃1/*N*. All green agents are passive spectators with score close to zero.

If for *β* = 1, *α* decreases from *α* = 1, then around *α* = 0.75 (for parameters of Eq (16)) there is a change in the final network structure. For *α* < 0.75 this structure is such that there emerge two leaders (*L*_1_ and *L*_2_), whose scores are approximately equal and much larger than the scores of all other agents; see Fig 11. They strongly drive each other (in the final state): *τ*_*L*_1_*L*_2__ = *τ*_*L*_2_*L*_1__ = 1). They drive *by turns* all other agents *k*; if *τ*_*kL*_1__(*t*) ≃ 1 and $\tau_{kL_{2}}\left(t\right)=O\left(1/N\right)$, then after a few time-steps *δ*, *L*_1_ and *L*_2_ interchange their roles: *τ*_*kL*_2__(*t* + *δ*) ≃ 1 and $\tau_{kL_{1}}\left(t+\delta \right)=O\left(1/N\right)$. For the noiseless situation (*η* = 0 in Eqs (18) and (19)) we get *δ* = 1, 2. If a weak noise is present see Eqs (18) and (19), *δ* becomes a random number.

All the other agents besides *L*_1_ and *L*_2_ are passive spectators without influence on anyone.

The transition from a single autocratic leader to the successive leadership takes place also for *β* = 0, but for *α* < 0.6 for parameters of Eq (16).

If *α* is close to zero (e.g., *α* ≤ 0.1 for *β* = 1 or *β* = 0), the relaxation to the stationary network structure is slow. For times *t* ∼ 30 − 40 there emerges a small (up to 10 agents for the overall number of agents 100) group of high-score leaders that influence each other and drive the remaining majority of agents without getting any sizable feedback from this majority. Only on much longer times, *t* ∼ 1000 − 2000, two leaders with the symmetric interaction emerge from within this group. Other agents from the high-score group move to the zero-score majority. Thus, there can be a larger group of leaders (more than two agents), but it is metastable.

## Conclusion

Leadership has been at the focus of many disciplines: history (many biographies are about leaders), political science (governance structure and function), philosophy (principles of good versus bad leadership), management science (leadership practices), social psychology (influence), communication research (leadership frequently goes via—and is about—communication), and complex systems [1, 2]. But we lacked a tractable model that can provide a theoretical laboratory for describing and studying leadership scenarios.

We studied a formal, mathematical model for the emergence of opinion leader in a collective of agents that are modeled via threshold elements (neurons) living on a network. Agents can activate and influence their neighbors via the network possibly making them active as well. Weights of the network links are dynamic. They account for the importance given by the agent to a specific link. The behavior rules of the agents are summarized as follows; cf. Table 1.

– The overall influence on an agent from its neighbors (i.e. its attention) is limited.
– An agent re-distributes its attention via changing the link weights such that more active agents get more attention. The conservatism of the agent during this process is described by a parameter *α* ≥ 0; see Eq (6). For *α* → 0 the agent tends to revise even those links that were once deemed to be unimportant.
– There is a possibility of involving into the model an agent’s credibility score, a dynamic variable that grows whenever the agent actively influences its neighbors; otherwise the score decays. The score couples to attention such that agents tend to be influences by their higher-score neighbors. The extent to which they account for their existing score is modeled by a parameter *β* = 0, 1; see Eq (7). For *β* = 0 an agent does not compare its own score with the score of its neighbors.
– Agents may be subject to noise, i.e. they activate or deactivate randomly.

For this model we uncovered several types of leadership—depending on *α*, *β*, the noise, and the importance of credibility scores (see Table 2)—that do correspond to basic scenarios known from social psychology and from real life.

- The laissez-faire leader emerges under weakly-noisy dynamics without scores. Here, the overall activity is irregular, followers are influenced weakly, their mutual interaction is not suppressed, and autonomous sub-leaders with their followers are allowed. Some followers are shared between the leader and sub-leader. The leader and sub-leader(s) influence each other with comparable weights. The emergence of the laissez-faire leader takes a lengthy time, but it is a real leadership, since the overall activity decays (though not completely) after suppressing the leader. The replacement of the laissez-faire leader occurs spontaneously and also takes a lengthy time. An important aspect of the laissez-faire leadership is that its presence and its effect is visible only for sufficiently long times. We uncovered this scenario by looking at time-averages.
  Other leadership scenarios emerge after introducing the scores. This emergence takes much shorter time.
- A participative (democratic) leader does influence all agents strongly, hence it suppresses inter-follower interactions. Still this leader accepts feedback from the followers: it is active precisely due this feedback. If sub-leaders are allowed, they are delegates of the leader. The latter does not influence followers of the sub-leader, but strongly drives the sub-leader, whose back-influence on the leader is weak. The leader emerges due to its social capital (well-connectedness in the network). Initially its social capital is only slightly larger than for the others, but it is amplified dynamically due to credibility scores, and finally leads this agent to leadership. Within this scenario, no leader can be imposed externally, if the inter-agent coupling is sufficiently strong.
- The autocratic leadership scenario is realized for *β* = 0 (see Table 2), i.e. when the agents do not respect their credibility scores when re-distributing their attention. This point is somewhat unexpected, naively one would think that the neglect of one’s own score is a route to democracy. An autocratic leader (i.e., the highest score leader with the most followers) does not accept feedback from its followers; they are completely passive. Instead, the activity of the leader is supported either by symmetric interaction with a single agent (helper), or by one-way driving from a group of hidden (i.e., lower-score) agents. Hence, these agents are the real leaders. No simple criteria was found for predicting the autocratic leader from the initial state. In a sense, this leader is selected for random reasons—a point that is anticipated to be one of the main dangers of autocratic organizations [10]. The autocratic scenario is susceptible to perturbations of the initial state (in contrast to the participative leadership); i.e. for certain initial states the activity of the system ceases because no leader is established. One aspect of this susceptibility is that an autocratic leader may be always imposed externally, again in contrast to the participative leader. For non-conservative agents (*α* → 0), the model predicts a coalition of autocratic leaders. However, the coalition is meta-stable; for long times it reduces to a pair of autocratic leaders that symmetrically interact with each other, and by turn drive the remaining agents.

The above scenarios were established under concrete dynamic rules for the behavior of agents, but it is likely that they will emerge more generally, i.e., for other rules. A detailed understanding of this premise, as well as empiric validations of the model, are left for future work. Here we only stress that there are several directions via which such a validation can progress. Within the main direction, one can check possible leadership scenarios in various examples of social media. Here the main problem is that normally the real network of influences between agents is not known. However, several methods were developed recently to resolve this problem [25–29], and since all the ingredients of our model (e.g. the attention restriction *etc*) have their analogues for agents of social media, one can hope to find a reasonable classification of social media leaders that does resemble the one studied here (i.e. laissez-faire, participative, autocratic, weak and strong forms of hierarchy and of influence sharing, duumvirate *etc*).

There are few other directions along which one can attempt to validate the present model. Recently, there was an interesting discussion on the emergence of leaders in collective of robots [84]. Once it is understood from our model that rather complex leadership scenarios can emerge from simple behavioral rules, one can model these rules for robots and look at their emergent leadership behavior. Next, there is a large body of work devoted to various situations of decentralized control; see e.g. [85]. By their very definition such scenarios imply the absence of any leadership. However, our results hint that claims on the absence of leadership may in fact be overstated, and that laissez-faire leaders may be hidden in (at least) some scenarios of decentralized control.

## Supporting Information

S1 Data The file S1_Data.nb provides a simple numerical code (written in Mathematica) for simulating one of the leadership scenarios discussed in the main text. (NB)

## References

[1] *Encyclopedia of leadership*. Goethals GR, Sorenson GJ, Burns JM, editors. Thousand Oaks, California: Sage; 2004.

[2] Winkler I. Contemporary Leadership Theories. Berlin: Springer-Verlag; 2010.

[3] Katz E, Lazarsfeld PF. Personal Influence: The Part Played by People in the Flow of Mass Communication. Glencoe: Free Press; 1955.

[4] Beckman MD. Are your messages getting through? Journal of Marketing. 1967: 31, 34–38. 10.2307/1249027

[5] Troldahl VC. Public Opinion Quarterly. 1966: 23; 609–614.

[6] Burt RS. The social capital of opinion leaders. Annals of the American Academy of Political and Social Science. 1999: 566; 37–54. 10.1177/0002716299566001004

[7] Corey LG. People who claim to be opinion leaders: identifying their characteristics by self-report. Journal of Marketing. 1971: 35; 48–53. 10.2307/1250457

[8] Stogdill RM. Personal factors associated with leadership: A survey of the literature. Journal of Psychology. 1948: 25; 35–71. 10.1080/00223980.1948.9917362 18901913

[9] Valente TW, Davis RL. Accelerating the diffusion of innovations using opinion leaders. Annals of the American Academy of Political and Social Science. 1999: 566; 55–67. 10.1177/0002716299566001005

[10] Deutsch KW, Madow WG. A note on the appearance of wisdom in large bureaucratic organizations. Behavioral Science. 1961: 6; 72–78. 10.1002/bs.3830060108 13722330

[11] Weimann G. On the Importance of Marginality: One More Step into the Two-Step Flow of Communication. American Sociological Review. 1982: 47; 764–777. 10.2307/2095212

[12] Hogg MA. Social Psychology of Leadership In: Kruglanski AW, Higgins EW, editors. Social Psychology: Handbook of Basic Principles. New York: Guilford Press; 2007.

[13] Lewin K, Lippitt R, White RK. Patterns of aggressive behavior in experimentally created social climates. Journal of Social Psychology. 1939: 10; 271–301. 10.1080/00224545.1939.9713366

[14] Fiedler FE. A theory of leadership effectiveness. New York: McGraw-Hill; 1967.

[15] Boccaletti S, Latora V, Moreno Y, Chavez M, Hwang DU. Complex networks: Structure and dynamics. Physics Reports. 2006: 424; 175–308. 10.1016/j.physrep.2005.10.009

[16] Blondel VD, Guillaume JL, Hendrickx JM, de Kerchove C, Lambiotte R. Local Leaders in Random Networks. Physical Review E. 2008: 77; 036114 10.1103/PhysRevE.77.036114 18517468

[17] Godreche S, Grandclaude H, Luck JM. Statistics of leaders and lead changes in growing networks. Journal of Statistical Mechanics. 2010; P02001.

[18] Freeman LC. Centrality in social networks: conceptual clarification. Social Networks. 1979: 1; 215–239. 10.1016/0378-8733(78)90021-7

[19] Borgatti SP, Everett MG. Models of core-periphery structure. Social Networks. 1999: 21; 375–35. 10.1016/S0378-8733(99)00019-2

[20] Rombach MP, Porter MQ, Fowler JH, Mucha PJ. Core-Periphery Structure in Networks. SIAM Journal of Applied Mathematics. 2014: 74; 167–184. 10.1137/120881683

[21] Sole RV, Ferrer-Cancho R, Montoya JM, Valverde S. Selection, tinkering, and emergence in complex networks. Complexity. 2002: 8; 20–33. 10.1002/cplx.10055

[22] Venkatasubramanian V, Katare S, Patkar PR, Mu FP. Spontaneous emergecnce of complex optimal networks through evolutionary adaptation. Computers & Chemical Engineering. 2004: 28; 1789–1798. 10.1016/j.compchemeng.2004.02.028

[23] Cholvi V, Laderas V, Lopez L, Fernandez A. Self-adapting network topologies in congested scenarios. Physical Review E. 2005: 71; 035103(R) 10.1103/PhysRevE.71.035103 15903479

[24] Kianercy A, Galstyan A. Coevolutionary networks of reinforcement-learning agents. Physical Review E. 2013: 88; 012815 10.1103/PhysRevE.88.012815 23944526

[25] Huberman BA, Adamic LA. Information Dynamics in the Networked World Lecture Notes Physics. 2004: 650; 371–398. 10.1007/978-3-540-44485-5_17

[26] Huberman BA, Romero DM, Wu F. Social networks that matter: Twitter under the microscope. 2008. Preprint. Available: arXiv:0812.1045v1.

[27] Gomez-Rodriguez M, Leskovec J, Krause A. Inferring Networks of Diffusion and Influence. ACM Transactions on Knowledge Discovery from Data. 2012: 5; Article 21. 10.1145/2086737.2086741

[28] Ver Steeg G, Galstyan A. Information Transfer in Social Media. In: Proceedings of World Wide Web Conference (WWW). Lyon: France; 2012.

[29] Ver Steeg G, Galstyan A. Information-Theoretic Measures of Influence Based on Content Dynamics. In: Proceedings of WSDM’13. Rome: Italy; 2013.

[30] Colomer JM, Leadership games in collective action. Rationality and Society. 1995: 7; 225–246. 10.1177/1043463195007002008

[31] Simaan M, Cruz JB. On the Stackelberg strategy in nonzero-sum games. Journal of Optimization Theory and Applications. 1973: 11; 533–555. 10.1007/BF00935561

[32] Eguiluz V, Zimmermann M, Cela-Conde C, Miguel M. Cooperation and the emergence of role differentiation in the dynamics of social networks. American Journal of Sociology. 2005: 110, 977–1008. 10.1086/428716

[33] Anghel M, Toroczkai Z, Bassler KE, Korniss G. Competition driven network dynamics: Emergence of a scale-free leadership structure and collective efficiency. Physical Review Letters. 2004: 92; 058701 10.1103/PhysRevLett.92.058701 14995348

[34] Guttenberg N, Goldenfeld N. Emergence of heterogeneity and political organization in information exchange networks. Physical Review E. 2010: 81; 046111 10.1103/PhysRevE.81.046111 20481790

[35] Lipowski A, Lipowska D, Ferreira AL. Emergence of social structures via preferential selection. Physical Review E. 2014: 90: 032817 10.1103/PhysRevE.90.032817 25314491

[36] Van Vugt M. Evolutionary origins of leadership and followership Personality and Social Psychology Review. 2006: 10; 354–371. 10.1207/s15327957pspr1004_5 17201593

[37] Hazy JK. Computer models of leadership: Foundations for a new discipline or meaningless diversion? The Leadership Quarterly. 2007: 18; 391–410. 10.1016/j.leaqua.2007.04.007

[38] Bonabeau E, Theraulaz G, Deneubourg JL. Phase diagram of a model of self-organizing hierarchies. Physica A. 1995: 217, 373–392. 10.1016/0378-4371(95)00064-E

[39] Ben-Naim E, Redner S. Rank Statistics in Biological Evolution. Journal of Statistical Mechanics. 2005: L11002 10.1088/1742-5468/2005/11/L11002

[40] Castellano C, Fortunato S, Loreto V. Statistical physics of social dynamics. Reviews of Modern Physics. 2009: 81; 591–646. 10.1103/RevModPhys.81.591

[41] Rashevsky N. Mathematical Theory of Human Relations: An Approach to a Mathematical Biology of Social Phenomena. Bull. Amer. Math. Soc. 1949: 55; 722–724. 10.1090/S0002-9904-1949-09260-1

[42] Rashevsky N. Topology and Life: In Search of General Mathematical Principles in Biology and Sociology. Bull. Amer. Math. Soc. 1954: 16; 317–348.

[43] Rashevsky N. Some Remarks on Topological Biology. Bull. Amer. Math. Soc. 1955: 17; 207–218.

[44] Rashevsky N. Life, Information Theory, and Topology. Bull. Amer. Math. Soc. 1955: 17; 229–235

[45] Shvyrkov VB. Toward a psychophysiological theory of behavior. Advances in Psychology. 1985: 25; 47–71. 10.1016/S0166-4115(08)61596-4

[46] Emelyanov-Yaroslavsky LB. Potapov VI. Live neuron and optimal learning rule. Biological cybernetics. 1992: 67; 67–72. 10.1007/BF00201803 1606245

[47] Emelyanov-Yaroslavsky LB. Potapov VI Self-organization of day cycle and hierarchical associative memory in live neural network. Biological cybernetics. 1992: 67; 73–81. 10.1007/BF00201804 1606246

[48] Klopf AH. The hedonistic neuron: a theory for learning, memory and intelligence. New-York: Hemisphere Publishing Corporation; 1982.

[49] Nowak A, Vallacher RR, Burnstein E. Computational social psychology: A neural network approach to interpersonal dynamics In: Liebrand WBG, Nowak A, Hegselmann R, editors. Computer Modelling of Social Processes. London: Sage; 1998 pp 97–125.

[50] Vidal JM, Durfee EH. Multiagent systems In: Arbib MA, editor. The Handbook of Brain Theory and Neural Networks. Cambridge: MIT Press; 2003.

[51] Larson-Prior LJ, Parallels in Neural and Human Communication Networks In: Michelucci P, editor. Handbook of Human Computation. New York: Springer Science + Business Media; 2013.

[52] Piedrahita P, Borge-Holthoefer J, Moreno Y, Arenas A. Modeling self-sustained activity cascades in socio-technical networks. Europhysics letters. 2013: 104; 48004 10.1209/0295-5075/104/48004

[53] Allahverdyan AE, Ver Steeg G, Galstyan A. Memory-induced mechanism for self-sustaining activity in networks. Physical Review E. 2015: 92; 062824 10.1103/PhysRevE.92.062824 26764761

[54] Granovetter M. The strength of weak ties. American Journal of Sociology. 1978: 83; 1420–1433. 10.1086/226707

[55] Granovetter M, Soong R. Threshold models of diversity: Chinese restaurants, residential segregation, and the spiral of silence. Sociology and Methodology. 1988: 18; 69–104. 10.2307/271045

[56] Morris S. Contagion. The Review of Economic Studies. 2000: 67; 57–78. 10.1111/1467-937X.00121

[57] Durlauf SN. A framework for the study of individual behavior and social interactions. Sociological methodology. 2001: 31; 47–87. 10.1111/0081-1750.00089

[58] Galster GC, Quercia RG, Cortes A. Identifying neighborhood thresholds: An empirical exploration. Housing Policy Debate. 2000: 11; 701–732. 10.1080/10511482.2000.9521383

[59] Watts DJ. A simple model of global cascades on random networks. Proceedings of the National Academy of Sciences. 2002: 99; 5766–5771. 10.1073/pnas.082090499 PMC122850 16578874

[60] Dodds PS, Watts DJ. Universal behavior in a generalized model of contagion. Physical review letters. 2004: 92; 218701 10.1103/PhysRevLett.92.218701 15245323

[61] Galstyan A, Cohen P. Cascading dynamics in modular networks. Physical Review E. 2007: 75; 036109 10.1103/PhysRevE.75.036109 17500761

[62] J. Cheng J, L. A. Adamic LA, J. Kleinberg J, J. Leskovec J. Do Cascades Recur? 2016: WWW, 11–15, Montréal, Québec, Canada.

[63] Sorrentino RM, Boutillier RG. The effect of quantity and quality of verbal interaction on ratings of leadership ability. Journal of Experimental Social Psychology. 1975: 11; 403–411. 10.1016/0022-1031(75)90044-X

[64] Mullen B, Salas E, Driskell JE. Salience, motivation, and artifact as contributions to the relation between participation rate and leadership. Journal of Experimental Social Psychology. 1989: 25; 545–559. 10.1016/0022-1031(89)90005-X

[65] Peretto P. An introduction to the modelling of neuronal networks. Cambridge: Cambridge University Press; 1994.

[66] Vanvreeswijk C, Abbott LF. Self-sustained firing in populations of integrate-and-fire neurons. SIAM Journal on Applied Mathematics. 1993: 53; 253–267. 10.1137/0153015

[67] Usher M, Schuster HG, Niebur E. Dynamics of populations of integrate-and-fire neurons, partial synchronization and memory. Neural Computation. 1993: 5; 570–586. 10.1162/neco.1993.5.4.570

[68] Huberman B, Wu F. The Economics Of Attention: Maximizing User Value In Information-Rich Environments. Advances in Complex Systems. 2008: 11; 487–496. 10.1142/S0219525908001830

[69] Goncalves B, Perra N, Vespignani A. Modeling Users Activity on Twitter Networks: Validation of Dunbar’s Number. PLOS ONE. 2011: 6; e22656 10.1371/journal.pone.0022656 21826200 PMC3149601

[70] Hodas N, Lerman K. How limited visibility and divided attention constrain social contagion. In: Proc. ASE/IEEE Intl. Conf. on Social Computing (SocialComm); 2012.

[71] von der Malsburg C. Self-organization of orientation-sensitive cells in the striate cortex. Kybernetik. 1973: 14; 85–96. 10.1007/BF00288907 4786750

[72] Allahverdyan AE, Galstyan A. Opinion Dynamics with Confirmation Bias. PLoS ONE. 2014: 9; e99557 10.1371/journal.pone.0099557 25007078 PMC4090078

[73] Aronson E. The Social Animal. 10th revised edition New York: Palgrave Macmillan; 2007.

[74] Sutton RS, Barto AG. Towards a modern theory of adaptive network: expectation and prediction. Psychological Review. 1981: 88; 135–170. 10.1037/0033-295X.88.2.135 7291377

[75] Ito J, Kaneko K. Spontaneous structure formation in a network of chaotic units with variable connection strengths. Physical Review Letters. 2001: 88; 028701 10.1103/PhysRevLett.88.028701 11801043

[76] Bornholdt S, Rohl T. Self-organized critical neural networks. Physical Review E. 2003: 67; 066118 10.1103/PhysRevE.67.066118 16241315

[77] Allahverdyan AE, Petrosyan KG. Statistical networks emerging from link-node interactions. Europhysics Letters 2006: 75; 908 10.1209/epl/i2006-10212-8

[78] Caldarelli G, Capocci A, Garlaschelli D. A self-organized model for network evolution. The European Physical Journal B. 2008: 64; 585–591. 10.1140/epjb/e2008-00243-5

[79] Jost J, Kolwankar KM. Evolution of network structure by temporal learning. Physica A. 2009: 388; 1959–1966. 10.1016/j.physa.2008.12.073

[80] Caldarelli G, Capocci A, De Los Rios P, Munoz MA. Scale-free networks from varying vertex intrinsic fitness. Physical review letters. 2002: 89; 258702 10.1103/PhysRevLett.89.258702 12484927

[81] Li-Sheng Z, Wei-Feng G, Gang H, Yuan-Yuan M. Network dynamics and its relationships to topology and coupling structure in excitable complex networks. Chinese Physics B. 2014: 23; 108902 10.1088/1674-1056/23/10/108902

[82] Gross T, Blasius B. Adaptive coevolutionary networks: a review. Journal of the Royal Society Interface. 2008: 5; 259–271. 10.1098/rsif.2007.1229 PMC2405905 17971320

[83] Debanne D, Gahwiler BH, Thompson SM. Long-term synaptic plasticity between pairs of individual CA3 pyramidal cells in rat hippocampal slice cultures. The Journal of Physiology. 1998: 507; 237–247. 10.1111/j.1469-7793.1998.237bu.x 9490845 PMC2230782

[84] Pugliese F, Acerbi A, Marocco D. Emergence of Leadership in a Group of Autonomous Robots. PLoS ONE. 2015: 10; e0137234 10.1371/journal.pone.0137234 26340449 PMC4560398

[85] Greene MJ, Pinter-Wollman N, Gordon DM. Interactions with Combined Chemical Cues Inform Harvester Ant Foragers’ Decisions to Leave the Nest in Search of Food. PLoS One. 2013: 8; e52219 10.1371/journal.pone.0052219 23308106 PMC3540075

